# Complexity of dopamine metabolism

**DOI:** 10.1186/1478-811X-11-34

**Published:** 2013-05-17

**Authors:** Johannes Meiser, Daniel Weindl, Karsten Hiller

**Affiliations:** 1 Luxembourg Centre for Systems Biomedicine, University of Luxembourg, 7, avenue des Hauts-Fourneaux, L-4362 Esch-Belval, Luxembourg

**Keywords:** Parkinson’s disease, Metabolism, Metabolomics, Dopamine, Oxidative stress, Tyrosine hydroxylase, Tetrahydrobiopterine, Aromatic L-amino acid decarboxylase, Catecholamines

## Abstract

Parkinson’s disease (PD) coincides with a dramatic loss of dopaminergic neurons within the *substantia nigra*. A key player in the loss of dopaminergic neurons is oxidative stress. Dopamine (DA) metabolism itself is strongly linked to oxidative stress as its degradation generates reactive oxygen species (ROS) and DA oxidation can lead to endogenous neurotoxins whereas some DA derivatives show antioxidative effects. Therefore, DA metabolism is of special importance for neuronal redox-homeostasis and viability.

In this review we highlight different aspects of dopamine metabolism in the context of PD and neurodegeneration. Since most reviews focus only on single aspects of the DA system, we will give a broader overview by looking at DA biosynthesis, sequestration, degradation and oxidation chemistry at the metabolic level, as well as at the transcriptional, translational and posttranslational regulation of all enzymes involved. This is followed by a short overview of cellular models currently used in PD research. Finally, we will address the topic from a medical point of view which directly aims to encounter PD.

## Introduction

The age-related Parkinson’s disease (PD) is the most common neurodegenerative motor disorder in the world, affecting millions of elderly people. The motor symptoms of PD, such as rigidity, tremor or bradykinesia, are caused by the degeneration of dopaminergic neurons within the *substantia nigra pars compacta*. Despite intensive research over the past years, there is no cure for this disease and even diagnosis of PD is complicated due to a lack of reliable diagnostic tests.

There are sporadic and inheritable forms of PD. Sporadic PD is by far the most common, and thus represents the more pressing medical need. However, similarities in both forms have led to the assumption that there are common underlying molecular mechanisms [1,2].

Major causes of neurodegeneration are mitochondrial impairment and oxidative stress. In this context it is interesting to note that although the adult human brain constitutes only about 2% of body weight, it consumes about 20% of the body’s oxygen and glucose for the production of energy in the form of adenosine triphosphate (ATP) [3]. Thus, this organ is particularly exposed to the consequences of mitochondrial energy metabolism malfunction and its resulting injurious transition. In addition to these well known parameters, the catecholamine (CA) metabolism is a unique feature of catecholaminergic neurons and represents an additional source for reactive oxygen species (ROS) production. According to this prompted oxidative stress, brain tissue samples of *post mortem* PD patients comprise increased levels of lipid peroxidation in the *substantia nigra*[4]. Catecholamine metabolism might be especially crucial for cellular redox homeostasis and could be a trigger for ROS overload, i.e. ROS that can no longer be detoxified by the cell. To better understand the catecholamine metabolism and its consequences to cellular integrity, a systems approach on a metabolic level would be beneficial.

Systems biology and personalized medicine have become a fast growing field and have been more and more advanced especially in the light of high computing power, low cost sequencing opportunities and complex networks, underlying disease pathologies. Cellular regulation typically operates on four levels, besides regulation of genome, transcriptome and proteome the metabolome is the fourth level of regulation. Altered metabolic levels have in turn impact on the level of genome, transcriptome and proteome. Analyzing the metabolome means to make a metabolic snapshot of the cell, which is challenging because metabolism has turnover rates in the range of seconds.

Recent publications, that have been made possible by the advancement of new technologies, describe in detail the underlying molecular mechanisms favoring these metabolic changes. In terms of today’s research these advancements pushed our limits and opened new horizons. Key technologies are very sensitive mass spectrometers coupled to gas or liquid chromatography and stable isotope labeling [5,6]. The simultaneous measurement of several hundred metabolites in one single sample is no longer a challenge [7]. However, the key advancement in all large scale and “omics” analyses is the valuable readout of these large data sets, from their respective software packages [8]. In terms of metabolomics, this means identifying significantly deregulated metabolites, calculating enzyme activities, tracing the metabolic fate of single metabolites and to even identify unknown metabolites. These advancements can be observed in the field of cancer research, which has evolved tremendously over the last years [9]. Different examples nicely demonstrate the adaptation of cellular metabolism as an result of genetic reorganization and the impact of metabolism on cellular and systemic functionality [10,11].

Mining the literature of the last decade and looking for data related to DA metabolism or CA metabolism in general – also with respect to PD – we felt that this area of research is underrated, at least in the field of metabolism. Most research has been based on genetic studies, since several genes could be successfully linked to a PD phenotype. But we should not forget that most cases of PD are still idiopathic, rather than of genetic heritage. Therefore, additional causes for the loss of dopaminergic (DAergic) neurons over time, should exist. One key player for DAergic cell death might be the DA metabolism itself, which serves as a major source of intracellular ROS production. In this review we present a detailed overview over DA metabolism in the central nervous system, integrating molecular and biochemical aspects. We will refer to informative articles that go deeper into the individual topics.

## On the origin of dopamine research

DA was first prepared long before its importance as neurotransmitter was discovered. It was originally synthesized in 1910 because of the strong physiological effects, observed for other phenolic bases like epinephrine [12,13], but due to its comparatively low effect on arterial blood-pressure it was mostly overlooked. The first time DA was found to occur in an organism was as a pigment-building metabolite in the plant *Sarothamnus scoparius*[14]. Later on, it was found to be a substrate of aromatic amino acid decarboxylase (AADC) [15]; which could be isolated from sympathetic ganglia [16] and other animal tissues [17]. DA is also prevalent in invertebrates [18].

Initially DA was only assumed to be a precursor of the catecholic neurotransmitters epinephrine (E) and norepinephrine (NE) or considered to be an intermediate in tyrosine degradation [15]. It was only later that DA was recognized as an independent neurotransmitter [19,20]. It took some more time till the first DA receptor was discovered [21]. The Nobel Prize in medicine and physiology in 2000 was awarded to Arvid Carlsson together with Eric Kandel and Paul Greengard, for their research in the field of CAergic neurotransmission in the 1950s that lead to new techniques for DA measurement, and most importantly to the insight that DA was itself a neurotransmitter [22]. Quickly afterwards PD was associated with neostriatal DA depletion [23] which led to the first PD treatment with L-3,4-dihydroxyphenylalanine (DOPA, levodopa) [24] which is still used today. Other disorders have in the meanwhile been associated with DA metabolism or signalling, emphasizing the importance of a well balanced DA metabolism. In schizophrenic patients increased DA release is observed [25] and PD-like side effects can occur in schizophrenia treatment [26]. Deficient DA-signalling also plays a role in attention deficit hyperactivity disorder (ADHD) [27] and GTP cyclohydrolase 1 deficiency (see GTPCH section) which leads to another movement disorder named Segawa disease [28].

## Dopamine biosynthesis

**Figure 1:**
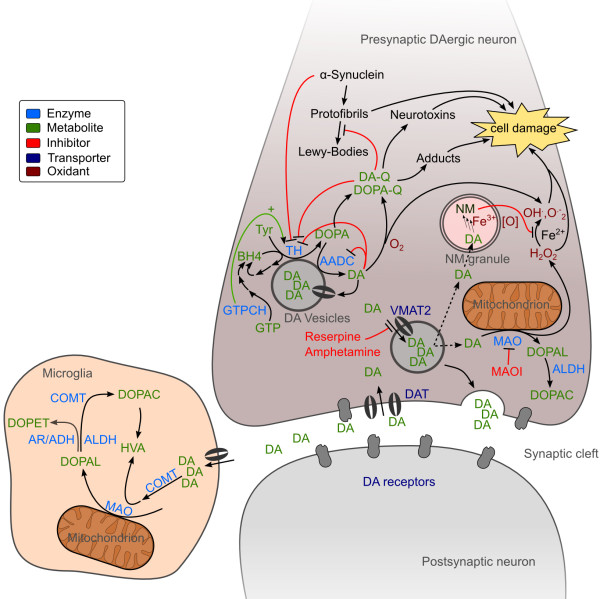
**Neuronal DA metabolism.** In the neurite of DAergic neurons, DA is synthesized by combined action of TH and AADC and imported into synaptic vesicles by VMAT2. DA leaking from the vesicles is deaminated by MAO. Upon neuronal excitation DA is released into the synaptic cleft for signal transduction. DA signaling stops by reimport to the presynaptic neuron and recycling or by import to surrounding cells and degradation by COMT, MAO, AR, ADH and ALDH. The main DA degradation products are DOPAC and HVA. In cytoplasmic vesicles NM is built of DA oxidation products and other components and can chelate iron. DA or DOPA can be oxidized to their corresponding reactive quinones (Q) that react further on to form a variety of partly neurotoxic compounds and protein adducts. These toxins and the ROS generated from DA deamination can cause cell damage and neurodegeneration. See text and Figures 2, 4 and 5 for further details and references.

**Figure 2:**
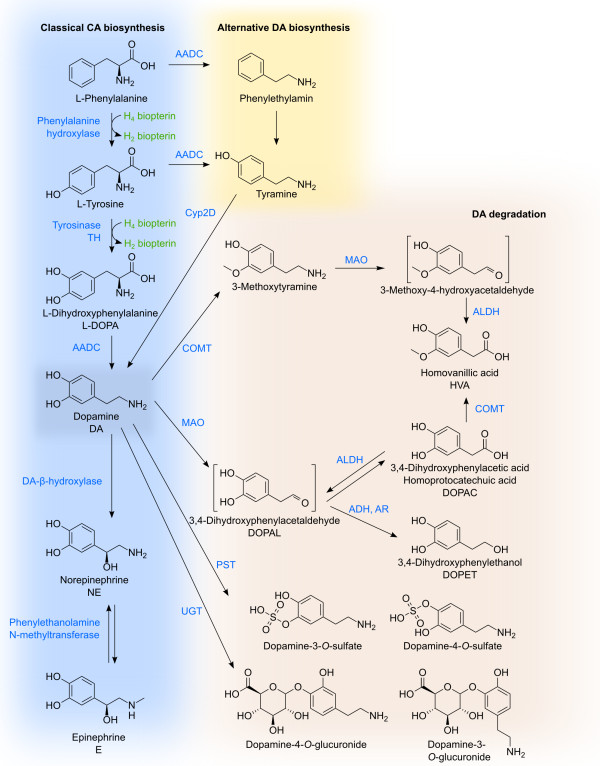
**DA biosynthesis and degradation.** The major pathway for DA biosynthesis starts at tyrosine or phenylalanine which can be hydroxylated by phenylalanine hydroxylase. Tyrosine is hydroxylated to form DOPA, now bearing the catechol moiety, by BH4-dependent tyrosine hydroxylase or alternatively by tyrosinase. Decarboxylation of DOPA by AADC leads then to DA. In another pathway for DA synthesis AADC action occurs before the hydroxylation at the aromatic ring. Tyramine is then oxidized by Cyp2D. Besides being a neurotransmitter itself, DA is also the precursor of epinephrine and norepinephrine. DA degradation is performed by COMT, MAO, ADH, ALDH and AR in variable order leading to DOPAC and HVA as the main endproducts. Phenolsulfotransferases and uridine diphosphoglucuronosyltransferases catalyze conjugation reactions with phosphate and glucuronic acid respectively. The relative contributions of the different enzymes are strongly species-, tissue- and celltype-dependent. The depicted reactions may occur in distinct compartments.

Although DA is an important neurotransmitter in the brain, a substantial part of the overall DA in the body is produced outside the brain by mesenteric organs [29]. We will focus here on DA production within the central nervous system (CNS). The classical pathway for DA biosynthesis was already postulated by Blaschko in 1939 [30]. The two-step biosynthesis of DA takes place in the cytosol of CAergic neurons and starts with the hydroxylation of L-tyrosine at the phenol ring by tyrosine hydroxylase (TH) to yield DOPA (Figures 1, 2). This oxidation is strongly regulated and depends on tetrahydrobiopterin (BH4) as a cofactor which is synthesized from guanosine triphosphate (GTP) by GTP cyclohydrolase (GTPCH). DOPA is then decarboxylated to DA by aromatic amino acid decarboxylase (AADC, also known as DOPA decarboxylase).

Besides this classical biosynthetic pathway, a cyto- chrome P450-mediated pathway was shown to exist in rat *in vivo*[31,32]. In this pathway decarboxylation precedes hydroxylation thus tyrosine is decarboxylated to tyramine which can then be hydroxylated by Cyp2D proteins (Figures 1, 2). Although the contribution to total DA synthesis seems to be low, it might become important under specific conditions [32].

Another possibility for DA biosynthesis is the tyrosinase catalyzed tyrosine hydroxylation and the subsequent DOPA uptake by CAergic neurons. Tyrosinase is normally involved in the biosynthesis of peripheral eumelanins and phaeomelanins [33], but for TH-negative mice this is the major source of CAs. Yet tyrosinase-lacking albino TH-negative mice still seem to have some source of CA [34]. It is not clear if this remaining DA is produced via the Cyp2D pathway or if other mechanisms still have to be discovered.

In CAergic neurons DA is readily sequestered into synaptic vesicles by secondary active transport via the vesicular monoamine transporter 2 (VMAT2) [35] (Figure 1). Inside these vesicles oxidation-prone DA is stabilized by the slightly acidic pH there [36]. This prevents oxidative stress in the cytosol [37]. Oxidative stress is further minimized by association of DA biosynthetic enzymes TH and AADC with VMAT2 [38]. Vesicular sequestration by VMAT2 can be irreversibly inhibited by the drug reserpine. Amphetamine and similar compounds inhibit VMAT2 directly and further collapse the proton gradient necessary for DA transport [35,39] (Figure 1).

To control DA homeostasis, the enzymes involved in DA synthesis – TH, GTPCH and AADC – play an important role to prevent excessive oxidative stress. In the following paragraphs we will present the underlying regulatory mechanisms that control enzyme activity of these proteins.

### Tyrosine hydroxylase

TH catalyzes the first step of DA biosynthesis and is strongly regulated. It constitutes, together with tryptophane hydroxylase and phenylalanine hydroxylase, the pterin-dependent aromatic amino acid monooxygenases [40,41]. TH consists of four identical subunits, each catalytically active and each of them requiring BH4, ferrous ion and O_2_ to oxidize tyrosine to DOPA [42].

Excellent in-depth reports of TH are available and should be consulted for further information [43,44]. Here we summarize the most important information to understand the regulation of TH activity and its importance for DA synthesis.

**Figure 3:**
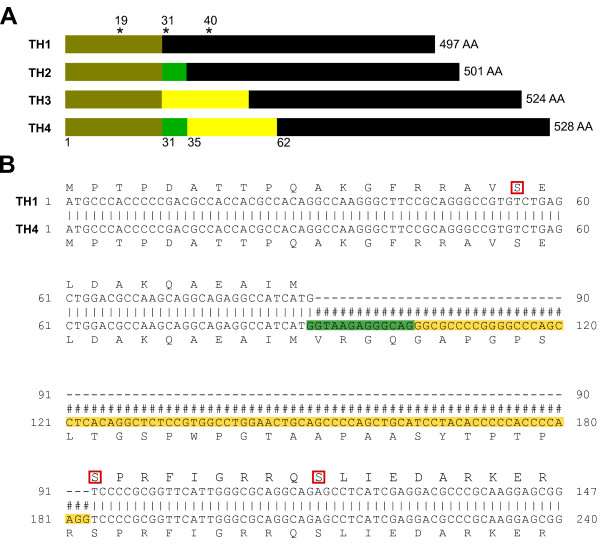
**Overview of TH isoforms.A)** Overview of the four human TH isoforms with their respective amino acid length. Asterisks indicate the position of the serines that are targeted by phosphorylation. Numbers on the bottom indicate amino acids located after a splice section. **B)** Alignment of TH1 and TH4 for illustration of the additional amino acids, present in the different isoforms. The numbers correspond to the nucleotide numbering.

TH is always coded by one single gene [45]. However, humans possess four TH isoforms due to alternative splicing in exon 2 [45-48] (Figure 3). Other primates have two isoforms and non-primate mammals have only one TH isoform [49,50]. Human TH1 (hTH1) is most similar to rat TH and hTH1 and hTH2 are predominantly expressed in human brain [47]. One should note, that the websites ensemble.org and NCBI show a different order and do not include TH2. In this manuscript we decided to stick to the nomenclature used in the literature (Figure 3).

The structure of all four isoforms is based on the same principle: one N-terminal regulatory domain ( ∼150AA), a central catalytic domain ( ∼300AA) and the C-terminal part, coding for a leucine zipper domain which is responsible for tetramer formation [51]. Loss of tetramer formation ability leads to a 70% drop of TH activity [52].

### Regulation of TH

TH is regulated on transcriptional [44,53-57] and post-transcriptional level [53] by covalent modifications, protein-protein-interaction and by allosteric regulation [43].

Synthesized CAs compete with the TH cofactor BH4 to bind the ferric ion at the catalytic site of TH [42,58-60]. Thus, high CA levels inhibit TH activity and thereby regulate its own intracellular concentrations via feedback regulation. The regulatory domain of hTH is targeted by phosphorylation at serine 19, 31 and 40 by various kinases, such as PKA, PKC CaMPKII, PKG, MPK, ERK which results in increased stability and/or activity [44]. Rat TH can also be phosphorylated at serine 8, but hTH has a threonine on this position instead. *In vivo*, depolarized cells increase their intracellular calcium concentrations via voltage sensitive calcium channels. Increase of calcium leads to the activation of different kinases, that in turn phosphorylate different serines on TH. Due to phosphorylation, the regulatory domain of TH undergoes a conformational change and dissociation of bound CA is facilitated. The phosphorylated version shows a sixfold higher dissociation rate compared to the non-phosphorylated form [59]. This is also demonstrated by Daubner *et al.* who generated phosphomimetic versions of TH by replacing Ser 40 by glutamate [60]. This version shows lowered inhibition by DA.

Phosphorylation of Ser 40 seems to have the strongest effect in terms of TH activation. Depending on the kinase and the position where TH is phosphorylated, the activity can increase up to 10 fold [60]. On the contrary, phosphorylation of Ser 19 seems to have two other purposes: a) it favors binding of regulatory 14-3-3 protein which in turn stabilizes TH [61-63] b) it facilitates Ser 40 phosphorylation (hierarchical phosphorylation) [64-66]. Phosphorylated Ser 31 results in a lowered *K*_m_ value for BH4 binding and a slight increase in activity, but this increase is only minor compared to Ser 40 phosphorylation [44]. Since Ser 40 seems to be the most important phosphorylation target in respect of activation it is interesting to note that only 5–11% of total TH proteins are phosphorylated *in vivo*[44,64,67].

To inactivate TH, there exist phosphatases (PP2A and PP2C) that can reverse the phosphorylation and might, therefore, function as deactivators [68-70]. TH can be inactivated by nitration, for example via reactive nitrogen species (peroxynitrite) or via *S*-thiolation on cysteine residues [71-74]. Regarding the stability of this enzyme, dephosphorylated TH versions are more stable compared to their phosphorylated counterparts. The explanation for this might be pretty simple, because DA levels have to be maintained at very defined levels and must not exceed thresholds of toxicity. Higher turnover rates of the active enzyme seem to be more feasible in order to better control how much DOPA is produced.

Besides serine 19, 31 and 40, arginine 37 and 38 have regulatory relevance for TH. Engineered enzymes with a deletion up to amino acid 39 [75] or arginine 37 and 38 replaced by glycine or glutamate showed higher activity due to favored BH4 affinity [76-78]. The authors speculated that these two amino acids might have important functions for the tertiary structure of the regulatory domain and enable DA mediated inhibition of TH [43].

A PEST domain has also been proposed for TH [79] and ubiquitylation of TH and associated proteasomal degradation was demonstrated [80,81]. However, we could not find any reference stating which lysine is targeted by ubiquitylation. UbPred an ubiquitylation site prediction tool [82] identified Lys 78 as the most likely target in TH4 (528AA). This would make sense as it lies within the regulatory N-terminal domain, which is exposed to the outside of the protein and would, therefore, be accessible for E3 ubiquitin ligase.

In addition to covalent modifications, TH stability is also controlled by interaction with other proteins (14–3-3, DJ-1, *α*-synuclein, VMAT-2, AADC, GTPCH) via the N-terminus of TH [38,43,61,62,83-85]; see also BH4 and GTPCH section. These interactions affect TH stability, activity and probably intracellular localization, which finally affects DA production.

One additional important factor regarding DA production and stability seems to be the intracellular O_2_ concentration. The O_2_ concentration in brain tissue is normally at 1–5%, whereas atmospheric oxygen levels are around 20%. Firstly, increased oxygen levels induce DA oxidation thus triggering the generation of ROS and secondly, the oxygen level influences TH protein abundance and activity [86,87].

It is important to mention that most biochemical *in vitro* studies have been performed with rat or other non-human TH. However, one should keep in mind that there are substantial differences between the species’ TH activities and their CA metabolism [60,88,89]. In summary, it is the N-terminal part of TH and especially its state of modification that plays an important role in protein stability and activity. In addition to active regulation of TH, the protein depends on the cofactor BH4 for catalysis. Regulation of BH4 synthesis and the role of GTPCH for DA production will be explained in the following section.

### BH4 and GTPCH

**Figure 4:**
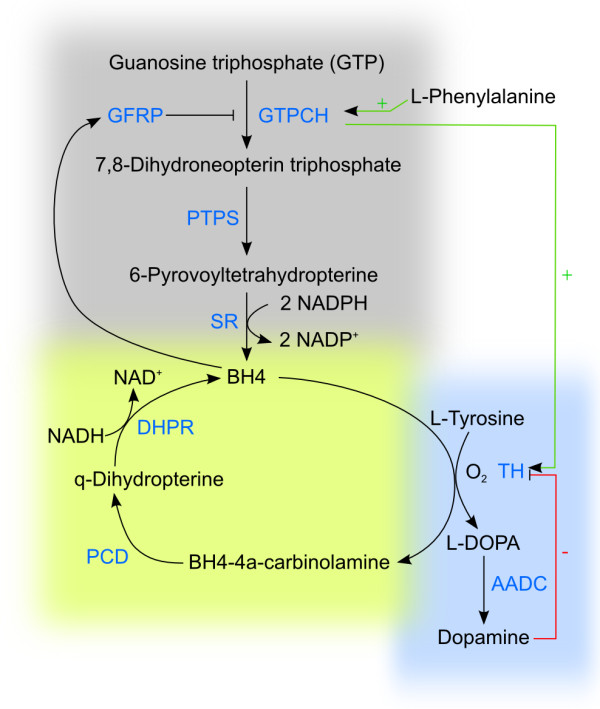
**Regulation of DA synthesis in dependency on BH4.** Dopamin synthesis relies on hydroxylation of phenylalanine, hydroxylation of tyrosine and decarboxylation of DOPA (blue box). The key enzyme tyrosine hydroxylase (TH) needs tetrahydrobiopterine (BH4) as a cofactor to catalyze the hydroxylation of tyrosine. Guanosine triphosphate (GTP) is the precursor for BH4 synthesis and GTP cyclohydrolase I is the key enzyme in this reaction (grey box). GTP cyclohydrolase I converts GTP into 7,8-dihydroneopterine triphosphate which is subsequently converted into 6-pyruvoyltetrahydropterine by PTPS. SR finally converts 6-pyruvoyltetrahydropterine into BH4. GTPCH is stimulated by Phenylalanine and repressed by high BH4 levels. in this case BH4 binds to the GTPCH feedback regulatory protein (GFRP). BH4 can be recycled via pterin-4a-carbinolamine dehydratase (PCD) and dihydropteridine reductase (DHPR) to maintain sufficient BH4 (yellow box).

6*R*-L-*erythro*-5,6,7,8-tetrahydrobiopterine (BH4) functions as a cofactor for the pterin-dependent aromatic amino acid monooxygenases and for NO synthase. BH4 can directly react with molecular oxygen to facilitate hydroxylation of the substrate. It is synthesized in three steps from GTP [90] (for review see Thöny *et al.*[91] and Werner *et al.*[92]). As an alternative to *de novo* synthesis of BH4, the cofactor can also be recycled via pterin-4a-carbinolamine dehydratase (PCD) and dihydropteridine reductase (DHPR) [91] (Figure 4). On the other hand, too high BH4 levels inhibit TH and are even toxic to the cell by inhibiting complex I and IV of the electron transport chain [93].

The first and rate-limiting reaction in BH4 production is catalyzed by the enzyme GTP cyclohydrolase I (GTPCH). GTPCH is coded by one gene and is built of six exons [94]. Alternative splicing yields at least three different splice variants, but only one version seems to be catalytically active. In addition, GTPCH is expressed in a tissue specific manner with especially high mRNA concentrations within serotonergic neurons. Results about GTPCH in CA producing neurons are controversial [95-98]. Dassesse *et al.* found relatively strong GTPCH immunoreactivity in the *substantia nigra* of rat brain [98]. Dominant as well as autosomal recessive GTPCH mutations have been reported and linked to DOPA responsive dystonia [99-102]. Other diseases associated with GTPCH or BH4 deficiency, respectively are hyperphenylalaninemia, cardiovascular disorders and phenylketonuria (PKU) [91,92,103,104].

Expression of GTPCH is regulated on transcriptional and post-transcriptional level. Administration of cAMP results in up-regulation of GTPCH gene expression. GTPCH activity is induced by phenylalanine and inhibited by BH4 via the GTPCH feedback regulatory protein (GFRP) [91,97,105,106]. In addition, phosphorylation of Ser 81 increases GTPCH activity [107-109].

### GTPCH-TH-interaction

Bowling *et al.*[83] could demonstrate that TH interacts with GTPCH and that this interaction depends on the phosphorylation of both. Interaction with TH prevented BH4-mediated inhibition of GTPCH, resulting in increased GTPCH and TH activities. These findings suggest that GTPCH activity is stimulated as long as TH is present in a phosphorylated (thus itself active) state and therefore DA production is also dependent on GTPCH. Experiments in *Drosophila melanogaster* showed that administration of BH4 could not restore TH activity in flies with mutated GTPCH versions. The authors assume that full TH activity depends on the interaction of TH with GTPCH [110]. These results were also confirmed by Bowling *et al.*[83], who showed that addition of GTPCH to TH increased *V*_*max*_ of TH. Interestingly, they also found a functional explanation for the phenomenon that high BH4 concentrations inhibit TH activity as previously reported [47] and that only physiological concentrations of 25–100 *μ*M increased TH activity. Others report that concentrations of 10 *μ*M have activating effects on TH [68]. However, there is a common agreement that the BH4 level has to be balanced. The concept is, that only a certain concentration of BH4 molecules results in active TH, because too high concentrations block GTPCH on the N-terminal part and prevent thereby the interaction with TH. Too low concentrations will be limiting due to lacking cofactor molecules for TH. In summary, TH needs both, the cofactor BH4 and the interaction partner GTPCH for functionality.

Although TH interaction with GTPCH prevents feedback regulation of GTPCH by its end product BH4, TH can still be inhibited by DA, even in the presence of GTPCH. This is based on the way these two enzymes undergo complex formation and the resulting three-dimensional structure [83]. These findings further advocate the complex underlying regulatory mechanisms that control intracellular DA levels.

### Aromatic amino acid decarboxylase

AADC was probably first described by Blaschko [30] and subsequently described by Schales and Schales [111] and Clark *et al.*[112]. Blaschko already asked the question whether AADC is specific to DOPA or if it can use other aromatic amino acids as substrate. Today we know that AADC uses pyridoxal phosphate (vitamin B6) as cofactor [113] and catalyzes the decarboxylation of several aromatic L-amino acids such as L-DOPA, L-tyrosine, L-tryptophane and L-histidine, thus being an important enzyme in the synthesis of different neurotransmitters and not exclusively specific to DOPA.

How CA biosynthesis in the human brain is regulated on the level of AADC is not completely clear [114]. AADC is regulated at transcriptional level and at post-translational level [115-117]. At transcriptional level AADC can be differentially expressed by alternative promoter usage and by alternative splicing [118]. At protein level AADC is regulated by phosphorylation [119] and DA receptor stimulation [117,120,121]. Based on the two different regulation types: transcriptional and post-translational regulation, AADC is regulated by a quick acting, short- term mechanism, via regulation of the protein activity and in a slower longer lasting regulation, by adapting the gene expression [115,116].

AADC activity is dependent on DA levels. By using the DA receptor antagonist cis-flupenthixol and haloperidol, an increase of striatal AADC activity could be detected [122,123]. DA receptor antagonists enhance the activity of AADC, whereas agonists are more likely to reduce activity [117,123]. In accordance to this, inhibition of MAO decreases AADC activity, implying that higher DA levels result in more DA bound to DA receptors [120,124]. Depletion of DA by reserpine treatment results in AADC activation [121]. Similar as TH, AADC is regulated in a species and tissue specific manner [115,116], which is even more reasonable for AADC, since it catalyzes the decarboxylation of different substrates in a wide range of tissues. Results about the kinetics are differing and seem to depend on the tissue, investigated [116].

Although TH is normally heavily regulated to control DA synthesis and AADC is not the rate limiting enzyme, AADC plays the key role in DA synthesis [125] if DOPA is administered as a drug to PD patients. In this case DOPA crosses the blood brain barrier via L-type amino acid transporters [126] to enter the endothelial cells from where it is sequestered to the neurons. Degradation of cytosolic DA by MAO and COMT as well as sequestration into vesicles via VMAT2 is even more important. Increased levels of DOPA not only have the potential to induce oxidative stress, but are also associated with schizophrenia [127]. In addition to DOPA administration, there are already ongoing clinical studies where AADC is targeted for gene therapy. More detailed research on human AADC would be beneficial to understand DA metabolism, also in respect of PD.

## Dopamine degradation

Upon excitation of DAergic neurons, the synaptic vesicles are emptied into the synaptic cleft (degranulation) to interact with the postsynaptic DA receptors or regulatory presynaptic DA autoreceptors [128,129]. To stop signaling, extracellular DA has to be removed from the synaptic cleft. It can either be recycled after reuptake by DAergic neurons or be degraded after uptake by glial cells.

Neuronal reuptake by DAT [130] is followed by sequestration into the synaptic storage vesicles by VMAT2. DA still accumulating in the cytosol, as a consequence of leakage from synaptic vesicles, is degraded by monoamine oxidase. Oxidative deamination by MAO produces hydrogen peroxide and the reactive 3,4-dihydroxyphenylacetaldehyde (DOPAL). This aldehyde can be inactivated by either reduction to the corresponding alcohol 3,4-dihydroxyphenylethanol (DOPET) or by further oxidation to the carboxylic acid 3,4-dihydroxyphenylacetic acid (DOPAC) by alcohol dehydrogenase (ADH) or aldehyde dehydrogenase (ALDH) respectively. Under normal conditions DOPAL is predominately oxidized to the corresponding carboxylic acid. While the reduction of DOPAL to DOPET occurs only to a very low extent, the deamination products of NE and E are mainly reduced to the alcohol [131].

Synaptic cleft DA is also taken up by surrounding glial cells. These cells readily degrade DA by MAO and also by catechol-*O* methyl transferase (COMT). COMT transfers methyl groups from *S*-adenosylmethionine (SAM) to hydroxyl groups of various catecholic compounds [132,133]. 3-*O*-methylation of DOPAC by COMT leads to homovanilic acid (HVA), one of the main degradation products of DA. COMT operates in glial cells but there is no COMT activity in DAergic *nigro-striatal* neurons [134].

### Conjugation — Glucuronides and sulfates

DA and its metabolites can further undergo phase II conjugation reactions before excretion. *O*-Sulfatation and *O*-glucuronidation occur in both CNS and periphery [135-137].

Sulfate formation is catalyzed by phenolsulfotrans- ferases (PSTs) that transfer sulfate from 3’-phospho- adenosine-5’-phosphosulfate (PAPS) to phenolic hydroxyls. Both 3- and 4-sulfates occur, but the 3-sulfates are predominant [132] (Figure 2). There are big differences in the extent of sulfatation between different species [29]. In rats and especially dogs, but not in guinea pigs, there was substantial sulfatation observed after oral DA application which did not occur after intravenous application [138]. There are even differences in respect to different brain areas with higher degree of sulfatation in the hypothalamus and hippocampus, and a lower percentage in the striatum [136].

Glucuronidation is performed by ER-bound uridine diphosphoglucuronosyltransferases (UGTs) [139] transferring glucuronic acid from UDP-glucuronic acid to DA. DA-4-*O*-glucuronide and DA-3-*O*-glucuronide are formed in almost equal amounts, but no *N*-glucuronide was found (Figure 2). Of all the human UGTs, only UGT1A10 was found to have substantial affinity to DA [140], but there is no UGT1A10 expression in the brain [140] that could be responsible for the DA-glucuronides found there [135].

The main excretion products of DA found in human urine are HVA, DOPAC, their sulfates and glucuronides as well as DA conjugates [132,141]. In the brain DA-Conjugates seem to play only minor roles as in rat brain microdialysates DOPAC and HVA are the main metabolites by far [135]. There are varying reports concerning the ratio of conjugated metabolites to non-conjugated ones and ratio of sulfatation to glucuronidation of DA metabolites is not the same for all metabolites. For instance for DA glucuronidation predominates over sulfatation in mouse and rat brains [135], whereas DOPAC is mainly sulfated in human and rat brains [141].

### Monoamine oxidase

MAO is a key player in monoamine degradation and target of many therapeutic inhibitors (MAOI). It catalyzes the oxidative deamination of CAs to the corresponding aldehydes using flavin adenine dinucleotide (FAD) as a cofactor and generates hydrogen peroxide as a side product. There are two forms: MAO-A and MAO-B, which are coded by two separate genes [142,143]. The enzymes are localized in the outer mitochondrial membrane and are found in both the CNS and the periphery. In the CNS MAO is present in neurons, microglia cells and astrocytes. *Substantia nigral* neurons show comparatively low MAO presence compared other neurons or glial cells [144].

There are species specific differences in affinity of the two enzymes: although the *in vitro* affinity of both MAO types is the same, DA is mostly oxidized by MAO-B in human, but by MAO-A in rats [89]. However, MPTP, a synthetic compound causing PD-like symptoms [145] is oxidized by MAO-B in both rat and primates [146].

### Catechol-*O*-methyltransferase (COMT)

The Mg ^2+^-dependent COMT transfers activated methyl groups from SAM to catechol hydroxyl groups [132,133] (Figure 2). There are two isoforms of COMT coded by one single gene [147]. The soluble cytoplasmic form is present in glial cells and the periphery, but the rough ER-bound isoform M-COMT on the rough ER is prevalent in neurons. The latter one has a higher CA affinity and is mainly responsible for metabolism of CAs originating from DAergic and NEergic neurotransmission whereas the soluble S-COMT is more responsible for exogenous CAs [89]. COMT activity is highest in excretory organs such as liver and kidney, but is also present in the CNS where it is most abundant in microglia cells. COMT is less prevalent in neurons and astrocytes and was not at all detected in human DAergic *nigro-striatal* neurons [134].

### Metabolic differences

Metabolic differences between species, organs and tissues make elucidation of DA metabolism more complicated; the multitude of different models used make it hard to combine the different findings [88,131,132,138]. As an example, urinary metabolite measurements were sometimes used, making it hard to unravel neuronal DA metabolism as these samples contain a mixture of DA metabolites derived from all the different tissues with their different predominant metabolic reactions. In this context, it is also important to keep in mind that almost half of the DA found in the body is synthesized in the gastrointestinal tract [29].

## Catecholamines, oxidative stress and inflammation

### Dopamine oxidation and oxidative stress

**Figure 5:**
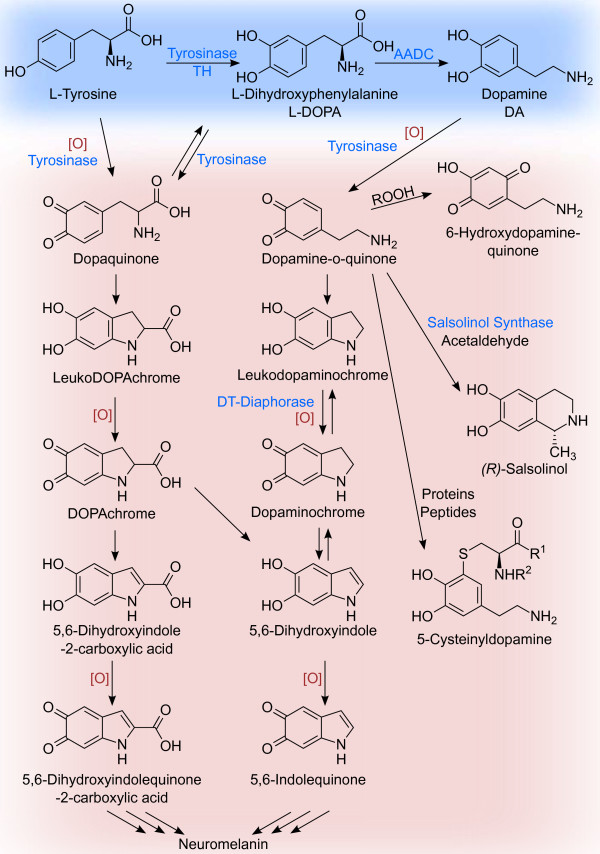
**CA oxidation products.** Catecholic compounds can be enzymatically or non-enzymatically oxidized to their corresponding quinones. These highly reactive compounds can undergo a multitude of different reactions, only a few are depicted here. Intramolecular cyclization and further oxidation of DOPA- and dopaminequinone lead to the precursors of neuromelanin. DA-quinone can react with hydrogen peroxide to 6-hydroxydopaminequinone, or with aldehydes to tetrahydroisoquinoline like salsolinol, both neurotoxic compounds. Cysteinylresidues of proteins or peptides readily react with DA-quinone to form 5-cysteinyl-DA-derivatives.

As described in the previous section, oxidative deamination of CAs by MAO generates hydrogen peroxide causing oxidative stress in CAergic neurons or CA-degrading cells. Besides this side-chain oxidation, DA as well as all other CAs are prone to oxidation at their electron-rich catechol moiety. DA and DOPA are easily oxidized enzymatically, by metal-catalysis (Fe ^3+^) [148] or even spontaneously, yielding the highly reactive electron-poor ortho-quinones DOPA-quinone and DA-quinone (Figure 5). CAs can be enzymatically oxidized by cyclooxygenases (COX, prostaglandin H synthase), tyrosinase and other enzymes [149,150]. With oxygen as the electron acceptor these reactions generate superoxide radical anions (O$O_{2}^{-\cdot }$). Both, quinones and ROS can react unspecifically with many cellular components altering their functionality and thus being potentially neurodegenerative. The DOPA-Q and DA-Q readily react with nucleophiles intra- and intermolecularly.

CA-quinones are central oxidation intermediates leading to a multitude of different products (Figure 5). Their amino group can attack the electrophilic quinone ring to form the cyclic aminochrome that tautomerizes to 5,6-dihydroxyindole a precursor for the neuronal pigment neuromelanin [151] (Figure 5). In the presence of iron DA-quinone can react further on to form the neurotoxin 6-hydroxydopamine [152]. DA-quinones are also precursors for the enzymatic formation of tetrahydroisoquinolines like salsolinol [151,153]. Salsolinol is an endogenous neurotoxin causing oxidative stress and mitochondrial damage by inhibition of the electron transport chain [153,154]. Additionally, salsolinol can heavily disturb CA metabolism by inhibition of TH, DA- *β*-hydroxylase, COMT and MAO [151].

Reaction of CA-quinones with e.g. thiol groups of amino acids and proteins lead to a variety of 5-cysteinyl-catechol derivatives. As cysteinyl residues of proteins are usually important for secondary structure and posttranslational modifications, their derivatization leads to impaired protein function. DAT and TH were already shown to be affected by DA-caused stress [73,155]. Conjugation of DA-quinone with glutathione limits the cell’s capability to deal with oxidative stress.

Another protein affected by DA oxidation products is *α*-synuclein, a major component of Lewy bodies, which are cytosolic inclusion bodies associated with PD [156,157]. *α*-Synuclein is a small protein ubiquitously present in the brain [158] and a negative regulator of DA biosynthesis due to interaction with TH [85]. DA or its derivatives as well as iron stabilize *α*-synuclein protofibrils thus preventing its inhibitory effect on DA synthesis, possibly leading to more oxidative stress [85]. More importantly, with PD-associated mutations of *α*-synuclein, these protofibrils seem to form membrane-permeabilizing pores probably leading to severe cellular dysfunction [159]. The mode of DA action is not clear here. As *α*-synuclein does not contain any cysteine residues, no cysteinyl derivatization can explain this effect [160].

The oxidation of the catechol moiety of CAs can be prevented by derivatization of its hydroxyl groups. *O*-Methylation by COMT not only inhibits oxidation of the compound itself, but additionally shows antioxidative effects by inhibition of metal-catalyzed ROS generation [161,162].

Oxidation chemistry of CAs and physiological implications have been thoroughly reviewed elsewhere [149-151,163].

### Neuromelanin

NM is a complex pigment found in specific brain regions, mostly in the *substantia nigra* and the *locus coerulus*. NM is built of DA-derivatives and contains 15% covalently bound amino acids and 20% adsorbed lipids [164]. It is not totally clear if enzyme-catalysis is needed in NM formation but at least iron is required, either as cofactor or alone [148]. Altough its structure is not totally clear, NM seems to be similar to the skin pigment melanin [165]. Studies on a synthetic DA-derived melanin suggests that it is not a covalently bound polymer but is kept together by *π*-stacking interactions [166].

NM is synthesized from non-vesicular DA. This could be demonstrated inhibition if its formation by VMAT2 overexpression [148]. NM is found in lysosome-like double membrane autophagic organelles within the cytoplasm [167], but no extracellular NM accumulation could be detected [150]. It is not clear at which stage DA, NM or the intermediates enter these NM granules. Overexpressed VMAT1 was reported to localize in endosomes of CHO cells [168] and could explain DA accumulation as NM precursor in endosomes or lysosomes. Additionally, with its lower affinity to DA compared to VMAT2 [35], VMAT1 could form a good secondary sink for excessive cytosolic DA. However, no VMAT1 could be found in NM granules [167] or in neuronal cells in general [169].

It is not totally clear if the polymer is degradable *in vivo* or not. At least there is no enzymatic degradation pathway known for NM, but it is sensitive to peroxidation *in vitro*[170]. As its formation is probably irreversible, excessive DA is sequestered effectively, reducing oxidative stress in the cytosol rendering NM synthesis neuroprotective [148].

Besides acting as a DA sink NM can bind transition metals, especially iron, preventing Fenton-type OH ^·^ radical generation (Fe(II) + H_2_O_2_ → Fe(III) + OH ^·^ + OH ^−^) and protect the cell from oxidative stress [171]. This is even more important for DAergic cells, as there is a higher ROS occurrence as compared to other cells.

Yet NM can turn detrimental depending e.g. on the iron load [172]. At some point the accumulation of metal ions within the polymer might become too high and turn detrimental. Oxidative stress might lead to NM degradation through peroxidation possibly leading to an release of previously captured metal ions or toxins, worsening the situation [173]. Neuronal cell death and subsequent release of NM might start a vicious circle of microglia activation and inflammation [174] causing more ROS stress and killing even more exhausted neurons [171].

### Oxidative stress, inflammation and neurodegeneration

Neuroinflammation in respect to PD is broad enough for its own review. Therefore, we refer to other reviews that nicely summarize this topic [175-178]. Here we will present some food for thought to illustrate the complexity of DA metabolism and its consequences.

As mentioned before, oxidative stress is part of DA metabolism due to its underlying chemistry. In general, oxidative stress is associated with many neuronal disorders such as Alzheimer’s Disease, PD and Schizophrenia [179]. On the other hand, ROS can be quenched by low-molecular antioxidants and antioxidant enzymes like superoxide dismutase (SOD), glutathione peroxidases (GPX) and catalase [180]. However, in the *substantia nigra* of PD patients, glutathione levels as well as the activities of SOD, catalase, and GPX have been shown to be decreased [151], rendering the cells more vulnerable to oxidative stress. Due to ROS overload, injurious effects such as lipid oxidation, uncoupling of the electron transport chain or DNA damage occur, which finally leads to cell death [181-184].

Oxidative stress signals and chemoattractants released by DAergic neurons result in activation of microglia cells and subsequent inflammatory reactions [176,185-188]. First observations for microglial activation in PD have been published in 1988 by McGeer *et al.* who analyzed tissue of the *substantia nigra* of PD patients *post mortem*[185]. Exposure to environmental toxins such as rotenone, MPTP and LPS lead to microglial activation [177]. Even years after MPTP exposure, activated microglia could still be detected [189,190]. Activation of microglial cells can also occur because of released NM from degenerating neurons as shown *in vitro*[174].

### Reactive nitrogen species (NOS) and regulation of DA levels

Upon microglial activation, intracellular NO production, synthesis of cytokins, inflammatory glycoproteins, chemokins and cell adhesion molecules are induced, resulting in adhesion of microglia cells to neurons. Chemoattractants released by degrading neurons promote these processes. Finally, microglia cells become phagocytic upon DAergic neurons [176]. NO can diffuse from activated microglia cells into DAergic neurons where it can react with superoxideanions (e.g. originating from the mitochondria) to peroxynitrite (${\text{NO}}_{3}^{-}$), a very potent oxidizing agent. In addition, production of hydrogen peroxide is increased, which further increases the level of ROS in neurons [177,191].

On the other hand, peroxynitrite can generate tyrosine nitrations in proteins, inhibiting TH activity [72,192]. NO-mediated repression of TH activity via *S*-thiolation on cysteine residues has also been reported [73,74] and is discussed in [43].

An additional regulatory mechanism to control intracellular ROS levels by adapting TH activity in dependence on the redox potential, is mediated by DJ-1, both on transcriptional and post-transcriptional level. DJ-1 upregulates TH transcription by altering the acetylation state of the TH promoter. DJ-1 silencing results in lowered TH expression and most probably less DA production [193]. Interestingly, the oxidation state of DJ-1 regulates its own activity and subsequently also TH expression [43]. Independent of the detailed molecular regulations that take place on TH, such modification could be a sensor for the intracellular redox level. When intracellular DA level rises, the level of oxidative stress and simultaneously peroxynitrite formation increases. Inhibition of TH would then inhibit DA formation to limit further ROS production. However, in the light of progressive PD such a repression of TH would be disadvantageous, because DA production will be further limited.

## Available cell models for research

Different cell lines are in use for research, related to the DAergic system. However, as described before there are strong species and tissue specific differences in regulating DA metabolism and DA synthesis. These differences make cell models of non-human or non-neuronal derivation not optimally suited for PD-related research.

PC12 cells [194] have been of great benefit in elucidating the kinetics of TH and its underlying biochemistry. However, PC12 cells are phaeochromacytoma cells of rat *adrenal medulla*, thus not originating from the CNS. In the original publication they are titled as “noradrenergic cells” [194]. Moreover, although they can be differentiated into non-dividing cells, they are still of cancerogeneous nature, and therefore, harbour a physiology far different from that of normal cells in tissue [195]. Another cell line in use is the MN9D line. This cell line originates from mice and was generated from a fusion of embryonic ventral mesencephalic and neuroblastoma cells. Differentiated MN9D cells were shown to express TH, voltage-activated sodium channels and to synthesize, harbour and release DA [196]. Although these cells can somehow mimic a DAergic neuron like phenotype, Rick and colleagues came to the conclusion that this cell line is not optimally suited as an *in vitro* model to study PD, because they do not mimic the electrophysiological properties of DA neurons [197]. If the cells are not electrical excitible, cell to cell communication may be lacking. Moreover, these cells are, as well as the PC12 cells, of non human origin.

SH-SY5Y is most probably one of the most frequently used cell line to mimic DAergic neurons. This line was subcloned from the original clone SK-N-SH, which was isolated from a neuroblastoma bone marrow biopsy [198-200]. Besides the fact that these cells are hard to cultivate and to differentiate into DAergic cells, these cells again originate from cancerogenous tissue. Most importantly there are reports that state that TH and AADC could not be detected in this cell line [160,201]. Xie *et al.* summarized in his review that “the SH-SY5Y cell line is not an ideal PD cell model” [201]. Balasooriya and Wimalsena characterised these cells physiologically and came to the conclusion that they are rather noradrenergic than DAergic [202].

LUHMES (LUnd Human MESencephalic) cells may be the most promising cell model currently available. They originate from 8-week-old fetal human ventral mesencephalic tissue, conditionally immortalized by introduction of *v-myc*[203,204]. These cells are human derived, of non cancerogenous origin and can be differentiated into postmitotic neurons, showing DAergic features, based on morphology, the expression of neuronal and DA specific marker genes, as well as neuron type like electrophysiological properties [204].

In moving towards personalized medicine, the future seems to lie in the use of induced pluripotent stem cells (iPS cells) [195]. In terms of a human-based model, the use of iPS cells differentiated into DAergic neurons is at the moment probably the most promising tool and is constantly under development [205-208]. Regarding embryonic stem cells (ESC), Cho and colleagues developed an efficient method to generate DAergic neurons from human ESC [209,210]. Their protocol yields in over 80 positive functional TH positive neurons. Transplantation of these cells into a parkinsonian rat model could demonstrate behavioral recovery [210]. However, ESC harbour the problem of availability and ethical problems, which in turn favors the use of iPS cells.

Compared to animal models, never changing arguments put the *in vitro* models into critizism. Cell models are monocultures: isolated, two-dimensional tissues, lacking a three-dimensional cell to cell communication as well as impulses from different cell types such as astrocytes or microglia. This makes other signals e.g. neurotransmitters like serotonin or GABA or signaling molecules like NO, missing in these cell models. Towards this end, attempts to mimic three dimensional like tissue structures [211] as well as co-cultures [212] are underway to encounter the proposed drawbacks and to develop models that are closer to *in vivo* reality.

## From the lab to clinical application

There is still no cure for PD and diagnosis is also not always easy. Different imaging methods are available and can be used for the classification of different idiopathic PD forms [213,214].

Treatments are available to alleviate the symptoms. As a medication, DOPA in combination with a peripherally-acting AADC inhibtor (carbidopa) is still the gold standard. Supplying DOPA as a DA precursor circumvents TH-deficiency but has major drawbacks. High DOPA dosages might become problematic in the light of highly toxic oxidation products which cause cell damage and inhibiting DAT and TH [73,155]. Moreover, high DOPA dosages could also be shown to reduce AADC activity over time and that DOPA “holidays” increased AADC activity [115,215]. Excessively supplied DOPA and its derivatives also cause problems when they undergo degradation by MAO and COMT. MAO-caused ROS use up the cell’s glutathione pool and can in turn cause oxidative damage. COMT-catalysed methylation of catechols potentially exhaust the cell’s methylation capacity [216]. This reaction depends on the universal methylation cofactor SAM, which is regenerated from homocysteine by cobalamine-dependent methylation from 5-methyltetrahydrofolate. DOPA administration was shown to lead to increased homocysteine levels and peripheral neuropathies [217-220], but this might be countered by coapplication of COMT inhibitors or folate and cobalamine [221]. Excessive DOPA treatment should therefore be carefully considered. Current techniques in drug delivery are moving towards extended drug release and non-oral administration which could help to circumvent fluctuating plasma levels as generated by current formulations [222].

Besides carbidopa and levodopa there are also drugs on the market or applied in clinical studies that target MAO B and COMT. Other trials target specifically the motor symptoms of PD by modulating glutamatergic, serotonergic or adrenergic systems. Different serotonin agonists for the treatment of PD symptoms are currently in clinical and preclinical trial [222]. Deep brain stimulation is currently used as an additional treatment option and shows amazing effects in diminishing the motor symptoms. The disadvantage of all therapies is the fact that symptoms are only attenuated for a limited amount of time.

Another promising idea is the use of iPS cells differentiated to DAergic neurons to replace the lost ones. These cells contain an identical genomic background as the patient but the risk of uncontrolled proliferation is currently not completely under control. However, attempts are on the way to attenuate these problems [223,224]. Alternative approaches aim to counter high levels of oxidative stress by using neuroprotective agents [225] or by using antiinflammatory drugs [191]. In this respect, nicotinic receptors are also promising targets for therapy. There exist reports showing that smoking leads to lowered DOPA dosages in PD patients. Furthermore, stimulation with a nicotinic agonist have resulted in increased amounts of TH protein [226-228].

A more recent wave of clinical phase I and II trials uses adeno-associated virus systems to deliver the important enzymes of DA metabolism - AADC, TH and GTPCH - into the affected brain region. However, by delivering AADC to the system [229,230], the treatment is only symptomatic, rather than targeting the roots of the disease. Engineering TH and GTPCH instead of AADC alone could help to improve the endogenous DA system. Such an attempt has already been made *in vitro*[231], in animal models [232] and is now also part of a phase I study [233]. An alternative gene therapy approach could be the use of engineered and more active TH versions, providing increased tyrosine hydroxylation rates and higher stability towards oxidative stress. However, this might be ethically more complicated and unwanted side effects must be minimized. For further details in state-of-the-art therapeutics and ongoing developments we recommend the article of Poewe *et al.*[222].

## Conclusions

The metabolism of DA sets DAergic neurons under constant oxidative stress. Therefore, DA homeostasis and ROS detoxification is of special importance. Syn- thesis and regulation of DA has been heavily investigated in the 20th century and many of its metabolic products as well as regulation of the synthesis en- zymes, have been unraveled in *in vitro* and *in vivo* experiments.

However, a detailed analysis of the DA metabolism and its consequences to the cellular integrity is important to understand disease mechanisms. It is especially important to distinguish between animal models and human based data. To investigate DA metabolism and degeneration of DAergic neurons as observed in PD, a human cell culture model harbouring the full metabolic pathway is indispensable. Although animal models have the advantage of having the whole organism with all the different tissues available, there are strong species specific differences in DA metabolism and regulation. For this reason, we feel that models of non-human and non-neuronal origin are only of limited use for research on human neurodegenerative diseases.

As presented here for DA metabolism and associated processes, there are intricate regulatory mechanisms in place for many biological pathways. To fully understand them, it is important to not only look at single aspects but to combine the different omics technologies with more classical fields of cell biology, enzymology and neuroanatomy to obtain a comprehensive systems level view.

In the case of PD, insights into DA metabolism, ROS detoxification as well as the consequences of DA-derived ROS-overload will help to understand the underlying problems of the disease and thus to develop new approaches to tackle this human burden.

## Abbreviations

AADC: Aromatic amino acid decarboxylase (DOPA decarboxylase); ADH: Alcohol dehydrogenase; ALDH: Aldehyde dehydrogenase; AMPH: Amphetamine; AR: Aldehyde reductase; ATP Adenosine triphosphate; BH4: 6R-L-erythro-5,6,7,8-tetrahydrobiopterin; CA: Catecholamine; CNS: Central nervous system; COMT: Catechol-O methyl transferase; COX: Cyclooxygenase; DA: Dopamine; DAT: Dopamine transporter; DOPAL: 3,4-dihydroxyphenylacetaldehyde; DOPAC: 3,4-dihydroxyphenylacetic acid; DOPET: 3,4-dihydroxyphenylethanol; E: Epinephrine; ER: Endoplasmic reticulum; GPX: Glutathione peroxidases; GTP: Guanosine triphosphate; GTPCH: GTP Cyclohydrolase; HVA: Homovanilic acid; iPS: Induced pluripotent stem cell; DOPA: L-3,4-dihydroxyphenylalanine; LPS: Lipopolysaccharide; MAO: Monoamine oxidase; MPTP: 1-methyl-4-phenyl-1,2,3,6-tetrahydropyridine; NE: Norepinephrine; NM: Neuromelanin; PAPS: 3’-phosphoadenosine-5’-phosphosulfate; PD: Parkinson’s disease; PGH: Prostaglandin H; PKM2: Pyruvate Kinase; ROS: Reactive oxygen species; SAM: S-adenosylmethionine; SOD: Superoxide dismutase; TH: Tyrosine hydroxylase; VMAT: Vesicular monoamine transporter.

## Competing interests

The authors declare that they have no competing interests.

## Authors’ contributions

JM and DW drafted and wrote the manuscript. KH contributed to the critical revision of the manuscript. DW prepared Figures 1, 2 and 5; JM and DW prepared Figures 3 and 4. All authors read and approved the final manuscript.
